# Influencing factors for postpartum depression in women with gestational diabetes mellitus

**DOI:** 10.3389/fendo.2024.1423127

**Published:** 2024-09-04

**Authors:** Jinfeng Tan, Yujing Xiong, Xiaohui Wang, Shiyao Wei, Chunqi Luo, Shaoming Huang, Yan Yang, Jinxia Chen, Jiebing Chen, Min Xu, Fengchun Wu

**Affiliations:** ^1^ Department of Obstetrics and Gynecology, the First Affiliated Hospital, Sun Yat-sen University, Guangdong Provincial Clinical Research Center for Obstetrical and Gynecological Diseases, Guangzhou, China; ^2^ Department of Reproductive Medicine, the First Affiliated Hospital, Sun Yat-sen University, Guangzhou, China; ^3^ Department of Oral Implantology, Hospital of Stomatology, Sun Yat-sen University, Guangzhou, China; ^4^ Research Department of The Sixth Affiliated Hospital, School of Medicine, South China University of Technology, Foshan, China; ^5^ Department of Internal Medicine, Zhongshan Hospital of Traditional Chinese Medicine, Zhongshan, China; ^6^ Department of Diagnostic Radiology, the First Affiliated Hospital, Sun Yat-sen University, Guangzhou, China; ^7^ The Affiliated Brain Hospital, Guangzhou Medical University, Guangdong Engineering Technology Research Center for Translational Medicine of Mental Disorders, Guangzhou, China

**Keywords:** postpartum depression, gestational diabetes mellitus, risk factor, protective factor, correlation

## Abstract

**Objective:**

It remains undefined about the association between gestational diabetes mellitus (GDM) and postpartum depression (PPD). Hence, a cross-sectional study was conducted to evaluate the association between GDM and PPD among pregnant women and to investigate the influencing factors for PPD.

**Methods:**

From June 2021 to June 2022, 205 parturients with GDM and 201 without GDM were included in the study as the GDM group and the control group, respectively. The collected data from the general information questionnaire and Self Rating Depression Scale (SDS) were statistically analyzed based on binomial logistic regression analyses and generalized linear mixed models (GLMMs).

**Results:**

Age at delivery, gestational age, glycosylated hemoglobin, triglyceride, SDS, and proportions of women who had a history of induced abortion or GDM were significantly different between the GDM group and control group (*P*<0.05). The incidence of PPD in the GDM group was significantly higher than that in the control group. The neonatal body weight and triglyceride in GDM women with PPD were significantly lower than those in GDM women without PPD (*P*<0.001). The univariate logistic regression analysis demonstrated that educational age was a protective factor, while glycosylated hemoglobin and GDM were risk factors for PPD. The multiple linear regression analysis revealed that neonatal body weight (*OR*=-0.904, 95%*CI*: -1.657 to -0.152, *P*=0.019) and educational age (*OR*=-0.166, 95%*CI*: -0.306 to -0.025, *P*=0.021) were protective factor, while GDM (*OR*=1.854, 95%*CI*: 1.027-2.681, *P*<0.0001) was a risk factor for PPD.

**Conclusion:**

GDM may be associated with PPD. Neonatal body weight and educational age were protective factors for PPD, and GDM was a risk factor for PPD. Therefore, more attention should be paid to the mental health status of women with GDM, especially those with lesser educational age and lower neonatal body weight.

## 1 Introduction

As a common medical complication of pregnancy, gestational diabetes mellitus (GDM) is defined as “any degree of glucose intolerance with its onset or first recognition during pregnancy” (1). The prevalence of GDM is estimated to range from 6.1 to 15.2% (2). This disease may increase the risk of adverse pregnancy outcomes, such as pre-eclampsia, large for gestational age (LGA), fetal loss, excess fetal adiposity, neonatal hypoglycemia, and higher rates of cesarean section. Due to the adverse maternal and neonatal effects of GDM, pregnant women with GDM may suffer more stress and worry, especially in those without proper control of plasma glucose (3). Further, it may also increase the risk of postpartum depression (PPD) and postpartum development of type 2 diabetes mellitus by up to 1.59 and 20 times, respectively (3–5).

Depression is a common mental disorder, with an estimated incidence of 5% in adults. This condition is characterized by a depressed mood or loss of pleasure or interest in enjoyable activities for a long time (6). PPD is defined as a major depressive episode within 4 weeks after childbirth (7). Mothers are diagnosed when they show either depression or anhedonia, as well as any five of the following symptoms: sleep disturbance, psychomotor agitation or retardation, feelings of worthlessness, fatigue, suicidal ideation or a suicide attempt, indecisiveness, and loss of appetite (7). The prevalence of PPD was estimated to be 17.22% (95% CI 16.00-18.51) worldwide (8).

The prevalence of depression is threefold and twofold higher in patients with type 1 diabetes mellitus and type 2 diabetes mellitus, respectively, compared with individuals without diabetes (9). It is suggested that diabetes may be associated with depression through several possible underlying mechanisms, such as inflammatory changes, hypothalamo-pituitary-adrenal (HPA) axis, serotonergic regulation, and psychobehavioral mechanisms (10). However, it remains undefined about the association between GDM and PPD. It has been confirmed that pregnant women with GDM have a 1.59 times (95% *CI*: 1.22-2.07, *P* = 0.001) higher incidence of PPD than those without GDM, and they may suffer more serious depression, suggesting that GDM may be associated with PPD (4, 5). Although the incidence of PPD in GDM women is higher than that in women without GDM [14.58%(95% *CI*: 4.2–24.9) vs. 9.06%(95% *CI*: 5.76–12.3)], the association is not significant in multivariate logistic regression analyses (*RR*= 1.56, 95% *CI*: 0.61–6.16; *P* = 0.35) (11). Other investigators have found that GDM is not significantly associated with prenatal depression or PPD (12). Given these facts, a cross-sectional study was conducted to investigate the association between GDM and PPD and analyze the influencing factors for PPD.

## 2 Materials and methods

### 2.1 Study participants

From June 2021 to June 2022, 205 parturients with a history of GDM in the Obstetric Clinic of the First Affiliated Hospital of Sun Yat-sen University were selected by convenient sampling and included as a GDM group, and other corresponding 201 healthy parturients were included as a control group. In the GDM group, the inclusion criteria included (1) parturients aged 18-49 years who had been diagnosed with GDM during pregnancy in our hospital; (2) parturients who participated in the 42-day postpartum follow-up program; (3) parturients who could complete the questionnaires independently; (4) parturients who were willing to participate in this study. The exclusion criteria included (1) parturients with a history of psychiatric disorders or neurological disorders (evaluated from verbal statement, medical records, and the Structured Clinical Interview for DSM-IV Disorders administered by professionally trained psychiatrists); (2) parturients with other endocrine diseases, hepatic dysfunction, or renal dysfunction; (3) parturients with cognitive dysfunction; (4) parturients with diabetes before pregnancy. For the control group, the inclusion criteria included (1) healthy parturients aged 18-49 years without GDM; (2) healthy parturients who participated in the 42-day postpartum follow-up program and were willing to participate in this study; (3) parturients who could complete the questionnaires independently. The exclusion criteria in the control group were the same as those for the GDM group.

### 2.2 Diagnosis of GDM

All respondents had received a 75-g oral glucose tolerance test (OGTT) at 24-28 weeks during pregnancy. The OGTT was performed in the morning after an overnight fast of at least 8 hours. The diagnosis of GDM was confirmed according to the GDM diagnosis criteria of China and the clinical practice recommendations of the American Diabetes Association (ADA) (13): the fasting plasma glucose (FPG) level ≥5.1 mmol/L (92 mg/dL), or the one-hour postprandial plasma glucose (1hPG) level ≥10.0 mmol/L (180 mg/dL), or the two-hour postprandial plasma glucose (2hPG) level ≥8.5 mmol/L (153 mg/dL). All data were extracted from medical records.

### 2.3 Methods

#### 2.3.1 General information questionnaire

A self-designed questionnaire was applied to collect the general information of respondents. Besides, their medical records were obtained, such as age at delivery (years), gestational age (weeks), body weight (kg) and height (cm) (to calculate the body mass index [BMI]), prenatal BMI, neonatal body weight, neonatal body height, monthly income of family, employment situation, educational age, payment, parity, history of abnormal pregnancy, history of induced abortion, history of spontaneous abortion, history of GDM, gravidity, parity, glycosylated hemoglobin, triglyceride, FPG, 1hPG, 2hPG, and maternal comorbidity or complications.

#### 2.3.2 Self-rating depression scale

SDS is a short self-administered questionnaire designed by Zung to quantify the depression severity of patients with depressive disorders (14). It consists of 20 items, which cover 4 common characteristics of depression (the pervasive effect, physiological equivalents, other disturbances, and psychomotor activities) rated by respondents according to their experience and feelings within the past week. Each item can be rated from 1 to 4 points (none or a little of the time, some of the time, good part of the time, and most of the time). The raw scores range from 20-80, which are multiplied by 1.25 to convert to index scores ranging from 25-100 (25-49: normal range; 50-59: mildly depressed; 60-69: moderately depressed; 70 and above: severely depressed) (15). Previous clinical studies have shown that using the SDS to measure depressive symptoms in PPD has yielded better results (16, 17). In this study, a score of 50 was used as the cut-off value to consider respondents as with or without depression. Further, these respondents with depression were categorized into mild, moderate, and severe levels according to their SDS scores. It has been reported that the split-half reliability of SDS is 0.73, the Cronbach’s alpha is 0.86, the sensitivity is 93%, and the specificity is 69%, respectively (18).

#### 2.3.3 Investigation method

All questionnaires were distributed to participants when they had returned to our hospital for maternal and neonatal examination 4 weeks after delivery under the requires of the 42-day postpartum follow-up program which they had agreed to participate. Subsequently, these questionnaires were retrieved. All the questions proposed by respondents were explained by trained on-site investigators, who had administered mental health investigation before, to ensure the questions were properly resolved and the questionnaires were fully completed. All subjects would undergo the Structured Clinical Interview for DSM-IV Disorders, and all subjects with PPD in the post-partum depression meet the Diagnostic and Statistical Manual of Mental Disorders Diagnostic and Statistical Manual of Mental Disorders (DSM-IV-TR) diagnostic criteria for postpartum depression, and those with psychiatric disorders that combine with other DSM-IV diagnostic criteria were excluded. All of the above criteria were completed by two professionally trained psychiatrists.

### 2.4 Statistical analysis

Double data entry was performed through Microsoft Excel 2013, and the data were statistically analyzed by SAS 3.0. Continuous data were presented as mean ± standard deviation (SD), and the statistical differences were assessed using the non-parametric Mann-Whitney U test. Categorical data was presented as frequencies and percentages, and the statistical differences were assessed using the chi-square test. The influencing factors for depression were investigated using binomial logistic regression analyses and generalized linear mixed models (GLMMs). The significant level was set as *P*<0.05.

## 3 Results

### 3.1 Characteristics of respondents

The general characteristics of all respondents in both groups (205 in the GDM group and 201 in the control group) are shown in **Table 1**. In 205 women with GDM, 203 cases received diet behavioral therapy, and 2 cases received insulin therapy. There were significant differences in the age at delivery, gestational age, glycosylated hemoglobin, triglyceride, SDS, and proportions of women who had a history of induced abortion and a history of GDM between the two groups (*P* < 0.05 or *P* < 0.001). Specifically, age at delivery, glycosylated hemoglobin, triglyceride, and SDS in the GDM group were significantly higher than those in the control group; while gestational age in the GDM group was significantly lower than that in the control group (*P* < 0.05). Besides, the proportions of women who had a history of induced abortion and had a history of GDM were higher in the GDM group compared with the control group (*P* < 0.05).

**Table 1:** Comparison of the basic data between the GDM group and the control group.

Variables	GDM group (n=205)	Control group (n=201)	*z* value	*P* value
	Mean ± SD	Mean ± SD		
Age at delivery (years)	33.93 ± 3.86	32.42 ± 4.14	**-3.692**	**<0.001**
Gestational age (weeks)	37.79 ± 2.74	38.69 ± 1.31	**-4.503**	**<0.001**
BMI (kg/m^2^)	23.31 ± 4.88	22.88 ± 2.58	-0.218	0.828
Prenatal BMI (kg/m^2^)	26.25 ± 5.38	26.62 ± 11.29	-0.088	0.930
Educational age (years)	15.78 ± 2.62	15.98 ± 2.35	-0.659	0.510
Glycosylated hemoglobin (%)	5.05 ± 0.47	4.83 ± 0.34	**-5.967**	**<0.001**
Triglyceride (mmol/L)	2.32 ± 0.94	2.04 ± 1.22	**-3.673**	**<0.001**
Neonatal body weight (kg)	3.06 ± 0.46	3.13 ± 0.37	-1.589	0.112
SDS	46.07 ± 9.73	40.52 ± 8.53	**-5.224**	**<0.001**
	GDM group	Control group	*χ^2^* value	*P* value
	*N* (%)	*N* (%)		
Employment situation				
Employed	173 (84.4)	176 (87.6)	0.846	0.358
Unemployed	32 (15.6)	25 (12.4)		
Gravidity				
1	84 (33.6)	101 (50.2)	5.236	0.073
2	57 (22.8)	56 (27.9)		
3	64 (25.6)	44 (21.9)		
Parity				
1	125 (61.3)	127 (63.2)	0.344	0.842
2	71 (34.8)	68 (33.8)		
3	8 (3.9)	6 (3.0)		
Children numbers				
1	121 (59.0)	123 (61.2)	0.222	0.895
2	77 (37.6)	72 (35.8)		
3	7 (3.4)	6 (3.0)		
Payment				
Without insurance	29 (14.1)	35 (17.4)	0.816	0.366
Medical insurance	176 (85.9)	166 (82.6)		
Monthly income of family				
CNY < 10 000	79 (38.5)	52 (25.9)	7.621	0.220
CNY 10 000 – 20 000	73 (35.6)	90 (44.8)		
CNY > 20 000	53 (25.9)	59 (29.4)		
History of abnormal pregnancy				
Yes	20 (9.8)	15 (7.5)	0.678	0.410
No	185 (90.2)	186 (92.5)		
History of induced abortion				
Yes	59 (28.8)	38 (18.9)	**5.443**	**0.020**
No	146 (71.2)	163 (81.1)		
History of spontaneous abortion				
Yes	28 (13.7)	28 (13.9)	0.006	0.937
No	177 (86.3)	173 (86.1)		
History of GDM				
Yes	60 (29.3)	37 (18.4)	**6.583**	**0.010**
No	145 (70.7)	164 (81.6)		

GDM, Gestational diabetes mellitus. Bold value means P<0.05.

### 3.2 PPD in both groups

Among 205 participants in the GDM group, 71 participants (34.6%) had PPD according to their SDS scores (≥50); while there were 35 participants (17.4%) with PPD in the control group (*n*=201). The difference was statistically significant (*P*<0.05). Among these 71 participants with PPD in the GDM group, there were 52, 19, and 0 with mild, moderate, and severe depression, respectively. In contrast, among these 35 participants with PPD in the control group, there were 34, 1, and 0 with mild, moderate, and severe depression, respectively. From another perspective, the proportion of GDM in parturients with depression was 67.0% (71/106), as compared with 44.7% (134/300) in those without depression, exhibiting a significant difference (*P*<0.05).

### 3.3 Certain characteristics between women with and without PPD in the GDM groups

As shown in **Table 2**, the neonatal body weight and triglyceride in GDM women with PPD were significantly lower than those in GDM women without PPD (*P*<0.001). The prenatal BMI, glycosylated hemoglobin, neonatal body height, FPG, 1hPG, and 2hPG between those GDM women with and without PPD were not significantly different (*P*>0.05).

**Table 2:** Certain characteristics between women with and without PPD in GDM groups mean ± SD.

Variables	GDM with PPD (n=71)	GDM without PPD (n=134)	*z* value	*P* value
Neonatal body weight (kg)	2.92 ± 0.51	3.09 ± 0.48	**-6.795**	**<0.001**
Glycosylated hemoglobin (%)	5.43 ± 0.48	5.35 ± 0.48	-1.463	0.144
Triglyceride (mmol/L)	1.17 ± 0.74	1.39 ± 1.03	**-3.467**	**<0.001**
Neonatal body height (cm)	47.92 ± 3.51	48.21 ± 2.48	-0.193	0.847
Prenatal BMI (kg/m^2^)	26.42 ± 3.85	26.19 ± 6.01	-1.712	0.087
FPG	4.51 ± 0.52	4.55 ± 0.50	-1.270	0.204
1hPG	10.06 ± 1.38	9.87 ± 1.56	-0.966	0.334
2hPG	8.83 ± 1.20	8.73 ± 1.61	-0.349	0.727

FPG, fasting plasma glucose; 1hPG, one-hour postprandial plasma glucose; 2hPG, two-hour postprandial plasma glucose. Bold value means P<0.05.

### 3.4 Influencing factors for depression

In this study, the univariate logistic regression analysis was performed, with depression as the dependent variable, general information as the independent variable, and the last category as a reference type. The results showed that educational age, glycosylated hemoglobin, parity, and GDM were associated with depression, with educational age and parity as protective factors, and glycosylated hemoglobin and GDM as risk factors. The odds ratio (*OR*) of educational age was 0.892 (95%*CI*: 0.800-0.996, *P*=0.042), revealing that the risk of depression decreased with an increase in educational age. The *OR* of glycosylated hemoglobin was 2.429 (95%*CI*: 1.251-4.716, *P*=0.009), indicating that the risk of depression increased with an increase in the glycosylated hemoglobin level. The *OR* of GDM was 6.701 (95%*CI*: 3.192-14.066, *P*<0.001), demonstrating that the risk of depression was higher in parturients with GDM than in those without GDM (see **Table 3**).

**Table 3:** Univariate logistic regression analysis results of depression disorders in the GDM group and the control group.

Factors	*B*	*S.E.*	*Wald*	*Sig.*	*OR* (95%*CI*)
Age at delivery (year)	0.016	0.035	0.220	0.639	1.016 (0.950,1.088)
Gestational age (weeks)	-0.060	0.053	1.283	0.257	0.942 (0.850,1.044)
BMI (kg/m^2^)	0.016	0.032	0.246	0.620	1.016 (0.955,1.081)
Prenatal BMI (kg/m^2^)	-0.002	0.018	0.016	0.898	0.998 (0.963,1.033)
Educational age (years)	-0.114	0.056	4.135	**0.042**	0.892 (0.800,0.996)
Glycosylated hemoglobin (%)	0.888	0.338	6.878	**0.009**	2.429 (1.251,4.716)
Triglyceride (mmol/L)	0.041	0.123	0.110	0.740	1.041 (0.819,1.324)
Gravidity			1.776	0.412	
1	0.301	0.348	0.751	0.386	1.352 (0.684,2.671)
2	-0.136	0.411	0.109	0.741	0.873 (0.390,1.954)
3	-	-	-	-	
Parity			8.824	**0.012**	0.350 (0.111,1.097)
1	-1.050	0.583	3.243	0.072	0.170 (0.049,0.590)
2	-1.771	0.634	7.800	0.005	
3	-	-	-	-	
Children numbers			5.317	0.070	
1	-0.849	0.626	1.838	0.175	0.428 (0.126,1.459)
2	-1.379	0.660	4.368	0.037	0.252 (0.069,0.918)
3	-	-	-	-	
Payment	-0.181	0.408	0.197	0.657	0.834 (0.375,1.855)
Monthly income of family			1.652	0.438	
CNY < 10 000	0.120	0.352	0.117	0.732	1.128 (0.566,2.249)
CNY 10 000 – 20 000	-0.305	0.359	0.720	0.396	0.737 (0.365,1.490)
CNY > 20 000	-	-	-	-	
History of abnormal pregnancy	-0.619	0.621	0.992	0.319	0.539 (0.159,1.820)
History of induced abortion	-0.097	0.339	0.081	0.776	0.908 (0.467,1.764)
History of spontaneous abortion	-0.177	0.432	0.169	0.681	0.838 (0.359,1.951)
History of GDM	0.427	0.311	1.879	0.170	1.532 (0.832,2.821)
GDM	1.902	0.378	25.280	**<0.001**	6.701 (3.192,14.066)

Bold value means P<0.05.

### 3.5 Multiple linear regression analysis results of the influencing factors for depression

#### 3.5.1 The association between neonatal body weight and depression

**Figure 1:**
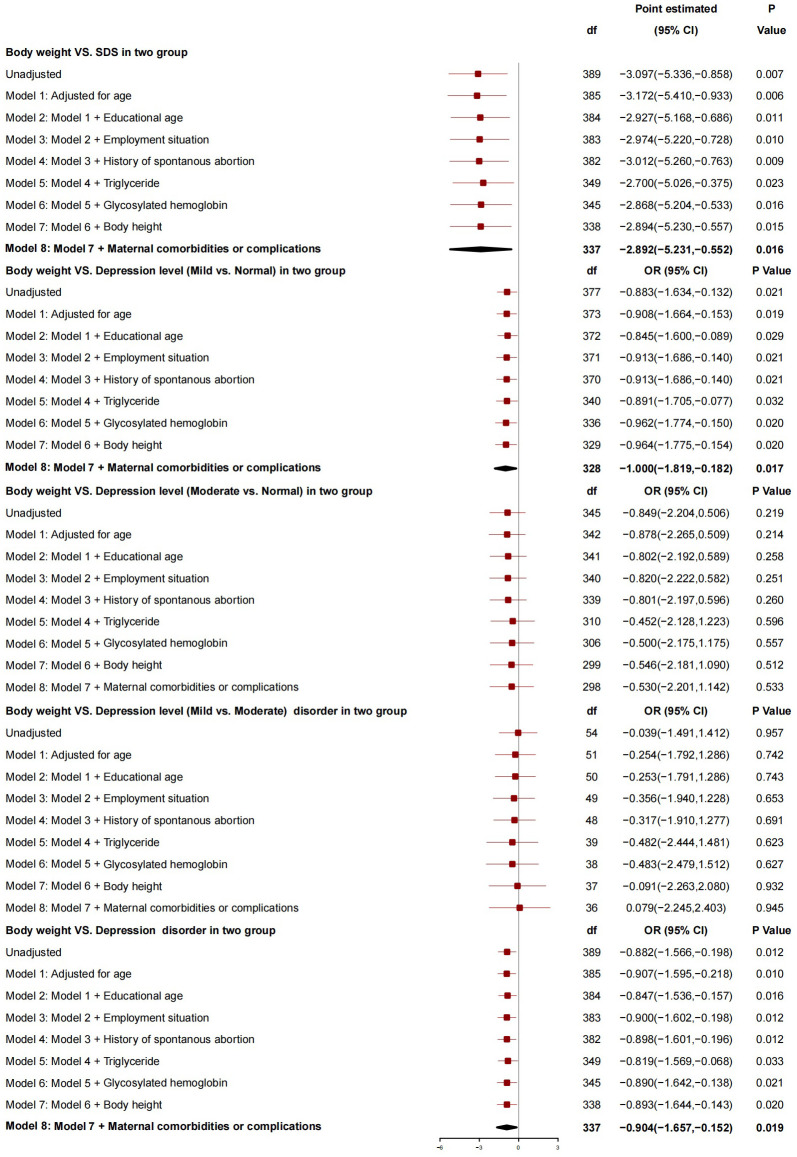
The association between neonatal body weight and depression.

After adjusting for covariates (age at delivery, educational age, employment situation, history of spontaneous abortion, triglyceride, glycosylated hemoglobin, neonatal body height, and maternal comorbidity and complications), the neonatal body weight was found to be associated with SDS. More specifically, the SDS decreased by 2.892 points with an increase of 1 kg in the neonatal body weight (95%*CI*: -5.231 to -0.552, *P*=0.016). Besides, the neonatal body weight was related to the severity of depression (mild *vs.* normal) [i.e., the risk of depressive severity (mild *vs.* normal) decreased by 1.000 with an increase of 1 kg in the neonatal body weight (95%*CI*: -1.819 to -0.182, *P*=0.017)]. However, the neonatal body weight was not related to the severity of depression (moderate *vs.* normal or mild *vs.* moderate). Overall, the neonatal body weight was a protective factor for depression (i.e., with an increase of 1 kg in the neonatal body weight, the risk of depression dropped by 0.904, 95%*CI*: -1.657 to -0.152, *P*=0.019) (**Figure 1**).

#### 3.5.2 The association between educational age and depression

**Figure 2:**
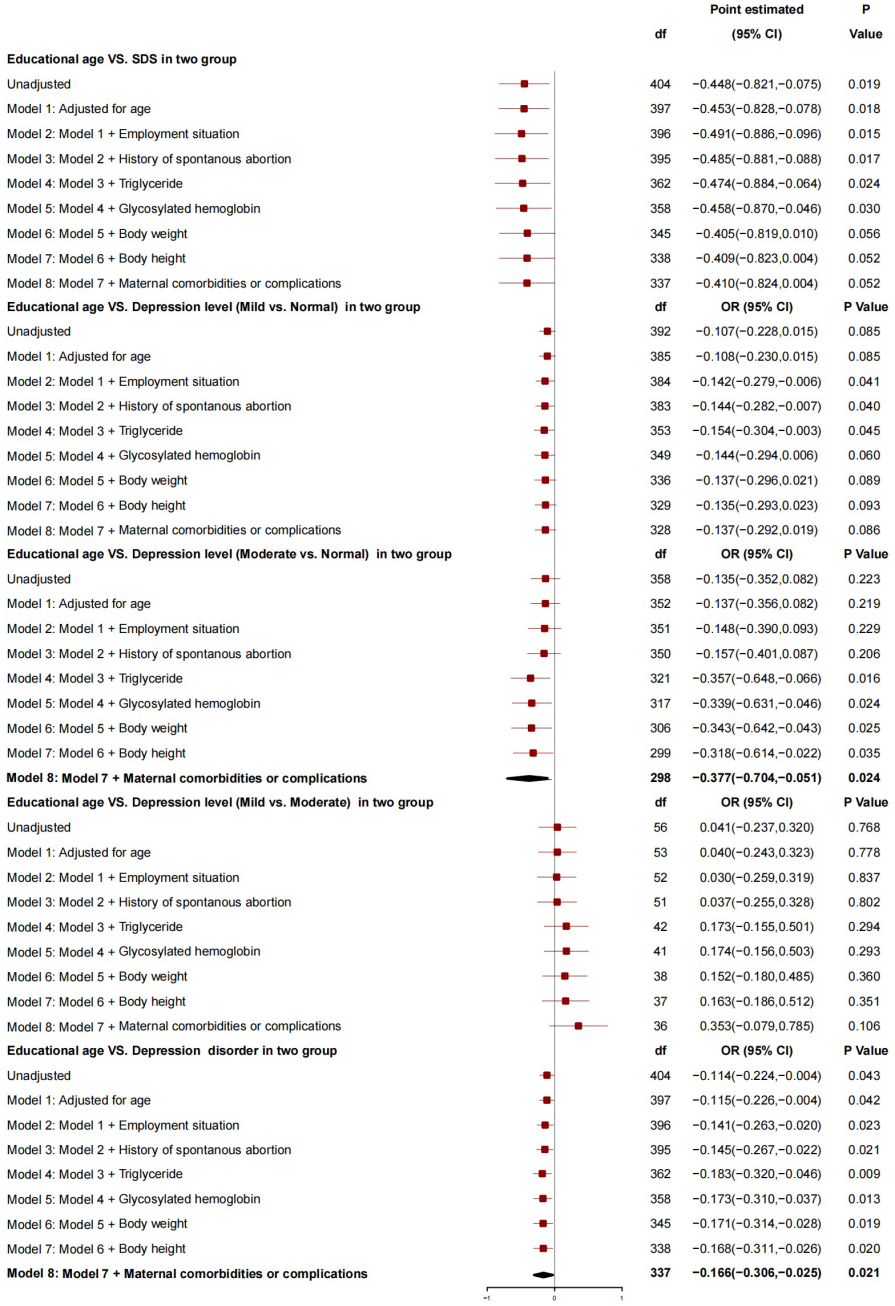
The association between educational age and depression.

After adjusting for covariates (age at delivery, employment situation, history of spontaneous abortion, triglyceride, glycosylated hemoglobin, neonatal body weight, neonatal body height, and maternal comorbidity and complications), educational age was found to have no association with SDS and the severity of depression (mild vs. normal or mild *vs.* moderate). However, it was associated with the severity of depression (moderate *vs.* normal) [i.e., the risk of depressive severity (moderate *vs.* normal) decreased by 0.377 with an increase of 1 year in educational age (95%*CI*: -0.704 to -0.051, *P*=0.024)]. Overall, educational age was a protective factor for depression [(i.e., the risk of depression decreased by 0.166 with an increase of 1 year in educational age (95%*CI*: -0.306 to -0.025, *P*=0.021)] (**Figure 2**).

#### 3.5.3 The association between GDM and depression

**Figure 3:**
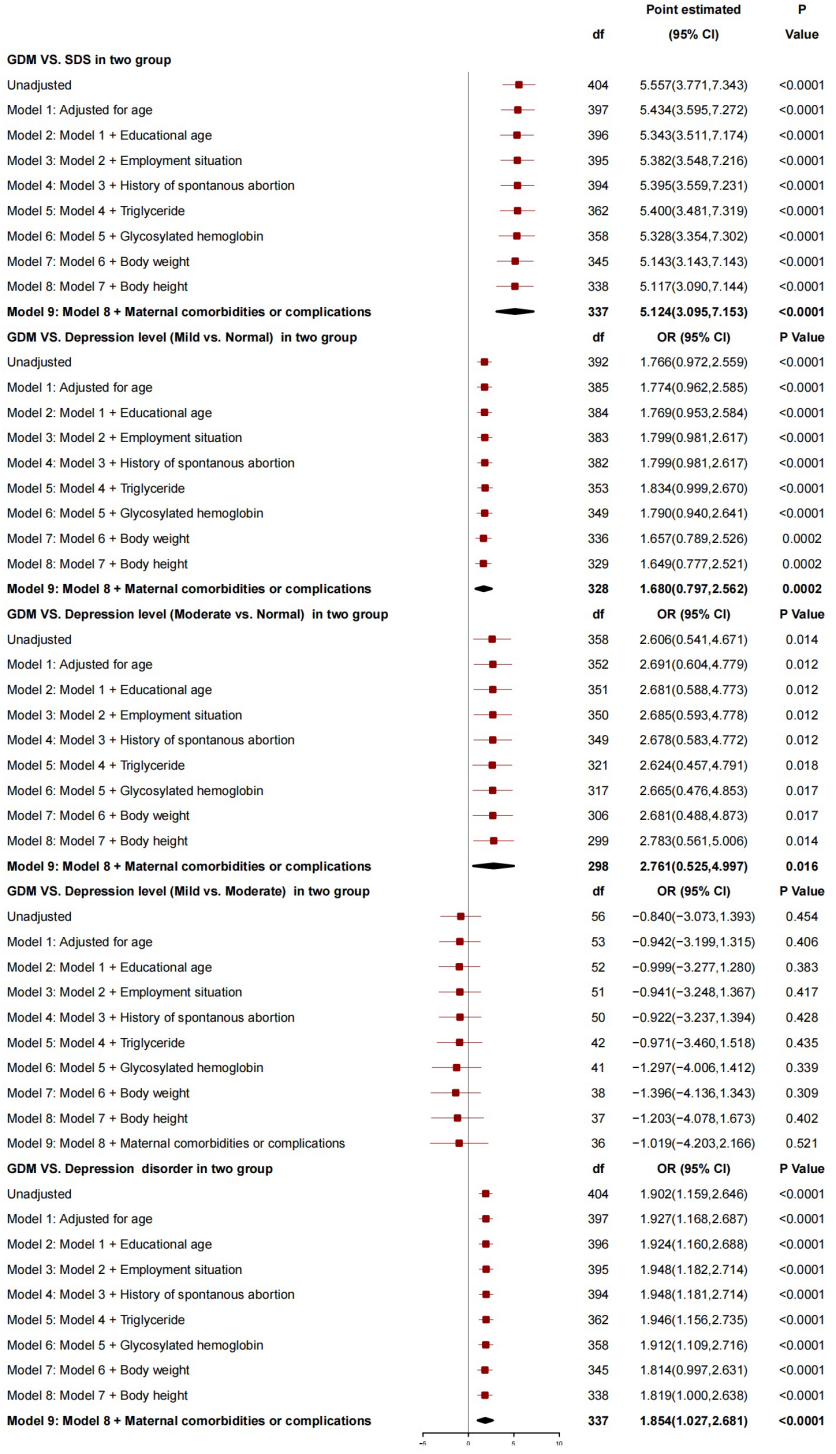
The association between GDM and depression.

After adjusting for age at delivery, educational age, employment situation, history of spontaneous abortion, triglyceride, glycosylated hemoglobin, neonatal body weight, neonatal body height, and maternal comorbidity and complications, GDM was found to be associated with SDS. The SDS in parturients with GDM was 5.124 points higher than that in those without GDM (95%*CI*: 3.095-7.153, *P*<0.0001). GDM was also associated with the severity of depression (mild *vs.* normal and moderate *vs*. normal). Compared with those without GDM, the risk of depressive severity (mild *vs.* normal and moderate *vs.* normal) in those with GDM increased by 1.680 (95%*CI*: 0.797-2.562, *P*=0.0002) and 2.761 (95%*CI*: 0.525-4.997, *P*=0.016), respectively. There was no association between GDM and the severity of depression (mild *vs.* moderate). Overall, GDM was a risk factor for depression, and the risk of depression in parturients with GDM increased by 1.854 (95%*CI*: 1.027-2.681, *P*<0.0001) compared with those without GDM (**Figure 3**).

## 4 Discussion

### 4.1 Neonatal body weight was a protective factor for depression

The neonatal body weight was positively correlated with the plasma glucose level in parturients with GDM, and the neonatal body weight increased by 44.9 g for each increase of SD in the FPG level in the third trimester of pregnancy (19). Women with GDM had 1.30 (95%*CI*: 1.23-1.38) times odds for LGA compared with those without GDM in southern China; however, the neonatal body weight showed a decreasing trend (from 3.224kg in 2012 to 3.134kg in 2021) in women with GDM (*P*<0.001), and the prevalence of macrosomia and LGA decreased from 5.1% to 3.0% and 11.8% to 7.7%, respectively, from 2012 to 2021, due to the diabetes self-management (20). In this study, 203 out of 205 women with GDM received diet behavioral therapy (only 2 cases received insulin therapy), and the results showed that their neonatal body weight was not significantly different from those without GDM, indicating the effectiveness of diet behavioral therapy. Our results also showed that the neonatal body weight in GDM women with PPD were significantly lower than those in GDM women without PPD, along with the triglyceride (*P*<0.001), suggesting that there may be existing excessive control over diet and behavior in order to reduce the risk of adverse pregnancy outcomes due to GDM. It was reported that intensive diabetes self-management was positively correlated with depression (21). Patients with diabetes mellitus tend to suffer more depressive disorders when undergoing intensive diabetes self-management (22). Only a few articles concerning one of the aspects of the question have been reported. One found that neonatal body weight was associated with maternal resilience (*β*= 370.2 ± 97.0, *P*=0.001) and life satisfaction (*β*= 423.3 ± 32.6, *P*=0.001) (23). It was reported in another study that low neonatal body weight was associated with PPD (AOR=5.69, 95% *CI*: 1.17-27.71) (24). Therefore, we conjecture that there may be an association between neonatal body weight and depression, and that the neonatal body weight may play an important role in linking GDM and PPD. We assumed that in GDM women following more strict diabetes self-management, lower neonatal body weight may suggest their overly done efforts in the control over diet and behavior, giving them a feeling of frustration and even regretion. Thinking that all the suffering they had been through under an intensive diabetes self-management means nothing may be a hard feeling for them. Future research should aim to control the intensity of diabetes self-management and neonatal body weight to better explore the association between GDM and PPD.

This study demonstrated that the risk of depression decreased with an increase in neonatal body weight, indicating neonatal body weight may be a protective factor for PPD, which was consistent with the findings of previous studies. However, it is necessary to further clarify their associations and the role of neonatal body weight in PPD or vice versa, which may be the topic of future studies.

### 4.2 GDM was a risk factor for PPD

The results of a systematic review and meta-analysis demonstrated that GDM was a risk factor for PPD (*OR* = 2.71, 95%*CI*: 1.78-4.14, *I^2^* = 0.0%) (25). Another study reported that the incidence of depression in women with GDM (19.0%) was significantly higher than that in those without GDM (9.3%) (21). Women with GDM had a nearly twofold greater risk (adjusted *HR*: 1.82, 95%*CI*: 1.28, 2.59) of depression compared with those without GDM according to a retrospective cohort study based on 58,400 mothers (25). Some possible mechanisms supporting the association between GDM and PPD had been proposed, but still not clear. One proposed that the stress from GDM and its adverse pregnancy outcomes, and the biochemical changes (i.e. catecholamine and serotonin levels) due to GDM may contribute to the development of PPD (26). The abnormal glucose metabolism in diabetes mellitus may be associated with the dysregulation of hypothalamic-pituitary-adrenal (HPA) axis and elevated cortisol levels, which in turn are related to PPD (27, 28). Moreover, the often increased cytokine-mediated inflammatory response and adipokine concentrations in women with GDM are supposed to be related to depression (29). Based on the experiment on GDM rat model, researchers found that GDM may disrupt both the tryptophan (Trp) metabolic pathway and the composition of the gut microbiota, which provided a putative physiological basis for PPD (30). Researchers have demonstrated in mice that postpartum HPA axis dysfunction is sufficient to induce postpartum depression, with maternal increased HPA axis excitability and glucocorticoid secretion (31). Change of serotonin (5-hydroxytryptamine, 5-HT) are the pathophysiology basis of mood disorders. 5-HT is mainly metabolized by tryptophan (Trp). When tryptophan metabolism is disordered, 5-HT synthesis is decreased, and kynurenine (Kyn) synthesis is increased. The disorder of Kyn/Trp ratio has been shown to be associated with depression and neuronal damage (32). During pregnancy, the levels of many hormones (including reproductive hormones such as progesterone, estradiol, thyroid-stimulating hormone, cortisol, cortitropin-releasing hormone or prolactin) increase, and then return to normal after partum. These dramatic changes of hormonal may influence endocrine diseases (such as diabetes) and mental health status (33). A prospective cohort study of 1,449 mothers found that high levels of FPG, 1hPG and 2hPG during pregnancy were associated with increased scores of PPD (34). But, based on the results of this study, FPG, 1hPG, and 2hPG between those GDM women with and without PPD were not significantly different (*P*>0.05), which was not consistent with previous reports (34). In this study, the incidence of depression among women with GDM was 34.6%, which was significantly higher than that (17.4%) among those without GDM (*P*<0.05). In the GDM group, the SDS was (46.07 ± 9.73), which was significantly higher than that in the control group (40.52 ± 8.53) (*P*<0.001). The univariate logistic regression analysis results revealed that the *OR* of GDM was 6.701 (95%*CI*: 3.192-14.066, *P*<0.001). Additionally, the risk of depression was higher in women with GDM than in those without GDM. Furthermore, after adjusting for covariates, the multiple linear regression analysis results showed that GDM was related to SDS and the severity of depression (mild *vs*. normal and moderate *vs*. normal). GDM was the risk factor for depression after delivery, and the risk was 1.854 times (95%*CI*: 1.027-2.681, *P*<0.0001) higher in women with GDM than in those without GDM. The results of this study were in line with those of previous reports, suggesting an association between GDM and PPD. However, association is not causality, and this cannot be identified based on the results of our study. Future researches should be performed to further elucidate the links and its possible mechanisms between GDM and PPD.

### 4.3 Educational age was a protective factor for depression

A cross-sectional study performed on 3,941 women confirmed that PPD was significantly independently related to the educational age of pregnant women, and parturients with depression showed a 38% higher possibility of having a lower educational age (35). It was also found that the prevalence of PPD in women with an educational age of less than 12 years was significantly higher than that in those with an educational age of more than 12 years (19.84% *vs*. 15.66, *P*<0.01), demonstrating an association between the educational age and PPD (8). Researchers proposed that the educational age was a significant predictor of PPD after delivery, and the risk of PPD increased with a decrease in the educational age (36). In this study, the OR of educational age was 0.892 (95%*CI*: 0.800-0.996, *P*=0.042). The univariate logistic regression analysis results confirmed that the risk of depression decreased with an increase in educational age. Although multiple linear regression analysis, which had adjusted for covariates, had revealed that educational age was not associated with SDS but associated with depressive severity (moderate vs. normal) (*P*=0.024). The risk of depressive severity (moderate vs. normal) dropped by 0.166 (95%*CI*: -0.306 to -0.025, *P*=0.021) with an increase of 1 year in educational age. This indicated educational age was a protective factor for depression, which was also consistent with previous reports. As a source of human capital, education may affect the general success of people and their effectiveness in pursuing fundamental ends that include emotional well-being (37). Generally, a higher education level can yield more mental resources, which in turn make people more resilient to strain or stresses, hence protecting them against depression (37).

There are three limitations in this study. (1) All participants were collected from one hospital, and hence the results may not be generalized to other regions or a larger population. (2) The study was conducted in a self-scored manner, so the objectivity of the findings may have been compromised despite prior internal consistency testing. Additionally, as an exploratory study, the use of a single SDS cut-off point in this study may have potential limitations that simplify the severity of depression, and future studies should be further analyzed in this regard. (3) The sample size was not large enough to draw a reliable conclusion about the correlation between GDM and PPD, and to derive conclusions about multiple influencing factors. Therefore, with a deeper analysis into the confounding variables, a multicenter study based on a larger sample size should be performed in the future, thus attempting to obtain a more reliable conclusion. Individual studies could also be performed for each influencing factor, to ensure that there is no unintentional bias that may be skewing the results.

## 5 Conclusion

The results of this study demonstrated that GDM was a risk factor for PPD, while maternal educational age and neonatal body weight were protective factors for PPD. In clinical practice, as for women with GDM, their mental status should be closely and regularly monitored and estimated, especially those with lesser educational age and lower estimated neonatal body weight, for early screening and timely management of PPD. Besides, proper control over blood glucose administered scientifically should be emphasized. Also, women with GDM could be counseled about the warning signs and symptoms of PPD. Further studies should be performed to explore the underlying mechanisms of the association between GDM and PPD, and the specific roles of educational age and neonatal body weight play in it.

## Data Availability

The raw data supporting the conclusions of this article will be made available by the authors, without undue reservation.
